# Synthesis, Crystal Structure and Antifungal Activity of (*E*)-1-(4-Methylbenzylidene)-4-(3-Isopropylphenyl) Thiosemicarbazone: Quantum Chemical and Experimental Studies

**DOI:** 10.3390/molecules29194702

**Published:** 2024-10-04

**Authors:** Haitao Ren, Fan Qi, Yuzhen Zhao, Abdelkader Labidi, Zongcheng Miao

**Affiliations:** 1 Technological Institute of Materials & Energy Science (TIMES), Xijing University, Xi’an 710123, China; 2 State Key Laboratory of Medicinal Chemical, College of Pharmacy, Nankai University, Tianjin 300071, China; 3 School of Environmental Science and Engineering, Shaanxi University of Science and Technology, Xi’an 710021, China; 4 School of Artificial Intelligence, Optics and Electronics (iOPEN), Northwestern Polytechnical University, Xi’an 710072, China

**Keywords:** thiosemicarbazones, crystal structure, quantum chemical calculation, Hirshfeld surface, antifungal activity

## Abstract

A novel (*E*)-1-(4-methylbenzylidene)-4-(3-isopropylphenyl) thiosemicarbazone was synthesized in a one-pot four-step synthetic route. Fourier transform infrared spectroscopy (FTIR), ^1^H and ^13^C nuclear magnetic resonances (NMR), single-crystal X-ray diffraction, and UV-visible absorption spectroscopy were utilized to confirm the successful preparation of the title compound. Single-crystal data indicated that the intramolecular hydrogen bond N(3)-H(3)···N(1) and intermolecular hydrogen bond N(2)-H(2)···S(1) (1 − x, 1 − y, 1 − z) existed in the crystal structure and packing of the title compound. Besides the covalent interaction, the non-covalent weak intramolecular hydrogen bond N(3)-H(3)···N(1) discussed by atoms in molecules (AIM) theory also functioned in maintaining the title compound’s crystal structure. The strong intermolecular hydrogen bond N(2)-H(2)···S(1) (1 − x, 1 − y, 1 − z) discussed by Hirshfeld surface analysis played a major role in maintaining the title compound’s crystal packing. The local maximum and minimum electrostatic potential of the title compound was predicted by electrostatic potential (ESP) analysis. The UV-visible spectra and HOMO-LUMO analysis revealed that the title compound has a low Δ*E*_HOMO–LUMO_ energy gap (3.86 eV), which implied its high chemical reactivity due to the easy occurrence of charge transfer interactions within the molecule. Molecular docking and in vitro antifungal assays evidenced that its antifungal activity is comparable to the reported *pyrimethanil*, indicating its usage as a potential candidate for future antifungal drugs.

## 1. Introduction

Thiosemicarbazone derivatives have attracted great attention in organic compound synthesis due to their potential applications in pharmaceutical synthesis as an intermediate and their multiple biological activities, such as anti-fungal [1], antiviral [2], antibacterial [3], antitumor [4], antiprotozoal [5], antiparasitic [6], etc. Typically, thiosemicarbazone derivatives are prepared utilizing the Mannich reaction between aldehyde or ketone with thiosemicarbazide [7], and the biological activity of thiosemicarbazone derivatives is related to substituents on aldehyde or amine [8]. Besides, thiosemicarbazone derivatives easily coordinate metal ions to form metal complexes, leading to a wide range of applications, such as enzyme inhibitors [9], fluorescent reagents [10], and antifungal [11] and electrochemistry materials [12]. Among them, fungi are one of the most extensively dispersed and biodiverse creatures, with an estimated 1.5 to 5.9 million species [13]. Over 8000 fungus species are known to cause illnesses in plants, with more than 300 appearing to be dangerous to people and animals [14,15]. The rise and global spread of highly infectious and transmittable pathogenic fungi pose a danger to human, animal, and ecological health and proliferation [16]. Given the need for new antifungal drugs, thiosemicarbazones have garnered attention in medicinal chemistry because of their high chemical diversity, intriguing biological uses, and significant pharmacological potential [17]. However, research on the use of thiosemicarbazone derivatives in antifungal drugs is still in its early stages [18]. Therefore, more efforts are desired to design novel thiosemicarbazone derivatives to promote their antifungal applications.

Computational chemistry is one of the most effective methods to explain the molecular structure and electronic properties of compounds in detail. In particular, the electronic properties obtained by quantum chemistry calculation could illustrate the interaction, reactive site, and charge transfer within molecules [19,20]. Spackman et al. [21,22] proposed the Hirshfeld surface, which mainly encodes the information of intermolecular interactions in crystal packing such as hydrogen bonds, π…π contact, van der Waals (vdW) force, etc. The corresponding 2D fingerprint plots were used to analyze the contributions of the intermolecular interactions that maintain the crystal packing stable [23,24]. Becke’s three-parameter hybrid exchange functionals with Lee–Parr correlation functionals (B3LYP) method has become the frequently used quantum mechanical modeling method to recognize the electronic structure of molecules [25]. Therefore, the chemical properties of thiosemicarbazone derivatives revealed by theoretical research based on quantum chemistry are of great significance in guiding their potential applications in biomedical fields.

In the current study, a (*E*)-1-(4-methylbenzylidene)-4-(3-isopropylphenyl) thiosemicarbazone was synthesized using a one-pot four-step route based on the previously reported method [26,27]. The difference between the current synthesis and previous reports is the addition of a sodium chloroacetate step. The structure of the title compound was characterized by FTIR, NMR, and single-crystal X-ray diffraction techniques. A Hirshfeld surface was employed to discuss and quantify the intermolecular interactions in the crystal packing of the title compound. The structure optimized at a B3LYP/6-31+G(d,p) basis set was utilized as the initial conformation of the quantum chemistry calculation. The AIM theory was applied to explain the strength of the bonding interaction of the title compound. The possible nucleophilic and electrophilic reactive sites of the title compound were predicted using ESP. UV-visible spectra and HOMO-LUMO analysis were combined to investigate the electronic transitions and reactivity of the title compound. Eventually, molecular docking in silico and disc diffusion assay in vitro were utilized to evaluate the antifungal activity of the title compound.

## 2. Results and Discussion

### 2.1. Chemical Synthesis

**Scheme 1:**
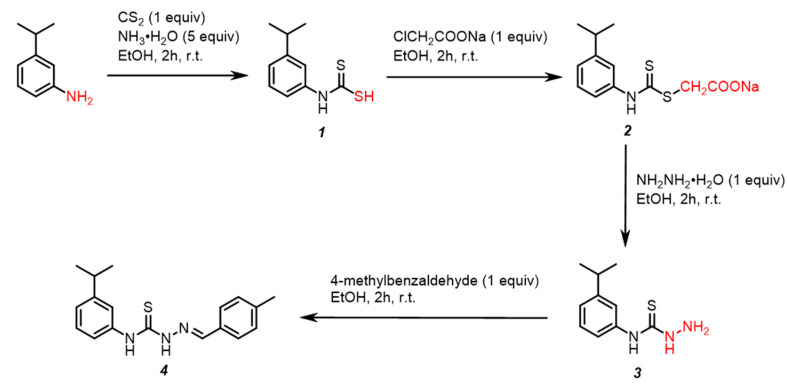
Synthetic route of the title compound.

First, we introduced the bulky isopropyl on the benzene ring to increase the solubility of the designed thiosemicarbazone by inhibiting intermolecular aggregation. Meanwhile, isopropyl, as an electron donor, improved biological activity via increasing electron density around the thiosemicarbazone unit (=N-NH-C(=S)-NH-). The synthesis of the (*E*)-1-(4-methylbenzylidene)-4-(3-isopropylphenyl) thiosemicarbazone references the previously reported one-pot four-step synthetic route [28], as depicted in Scheme 1. First, the ethanol solution of 3-isopropylaniline, 25% concentrated aqueous ammonia, and carbon disulfide was blended and stirred to form intermediate **1**, which is then reacted with sodium chloroacetate to obtain intermediate **2**. Subsequently, a nucleophilic substitution reaction occurred between intermediate **2** and 95% hydrazine hydrate to acquire key intermediate **3**. Finally, the title compound was obtained using a classical Mannich reaction of **3** and 4-methylbenzaldehyde in a moderate 43.4% yield. Furthermore, the FTIR spectrum of the title compound, as depicted in Figure S1, was recorded within the range of 4000*–*500 cm^−1^. Figures S2 and S3 show the ^1^H NMR and ^13^C NMR spectra of the title compound. According to the above spectral analysis, the basic skeleton of the title compound was initially confirmed.

### 2.2. Description of the Crystal Structure

**Figure 1:**
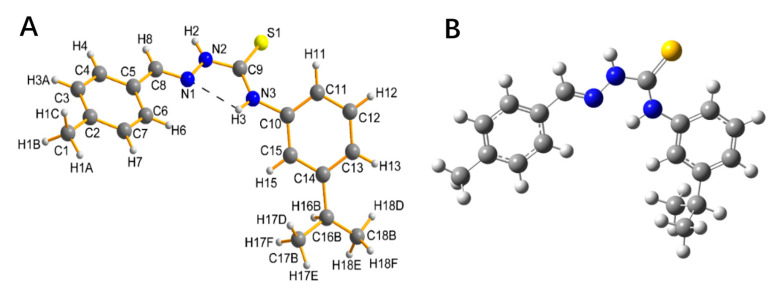
(**A**) Crystal structure and (**B**) optimized structure of the title compound. The black dotted line represents the intramolecular hydrogen bond.

The title compound formed the white block crystal. Based on single-crystal X-ray diffraction analysis, Table 1 summarizes detailed information on data collection and structural refinement. In addition, the crystal structure of the title compound (atomic labeling) is shown in Figure 1A. The optimized structure of the title compound calculated by the B3LYP/6-31+G(d,p) basis set in the gas is displayed in Figure 1B. The computation of wavenumbers, which produced positive values for every wavenumber obtained, verified the stability of the improved geometry.

Table 2 contains a list of certain experimental and theoretical geometrical parameters of the title compound, such as bond lengths and bond angles. Bond lengths N(1)-C(8) and S(1)-C(9) of the title compound, experimentally measured, are 1.269 and 1.668 Å, which show the characteristics of the typical double bond. There is a minor difference between bond lengths N(2)-C(9) (1.357 Å) and N(3)-C(9) (1.336 Å) in the experiment, and the delocalization of the electrons around the related atoms caused them to be smaller than average (1.472 Å). The experimental bond length N(2)-C(9) is in agreement with the theoretical value. The bond angles of C(17B)-C(16B)-C(18B), C(13)-C(14)-C(16B), C(4)-C(5)-C(8), and C(3)-C(2)-C(7), experimentally measured, are 123.0°, 119.1°, 119.9°, and 117.4°, respectively. The torsion angles of C(8)-N(1)-N(2)-C(9), N(1)-N(2)-C(9)-S(1), and N(1)-N(2)-C(9)-N(3), experimentally, are, in order, −179.0°, −177.3°, and 3.9°, which proves that the central skeleton (=N-N-C(S)-N-) of the title compound is almost coplanar. From the comparison, it is evident that the title compound’s experimental geometrical properties match the theoretical values.

**Figure 2:**
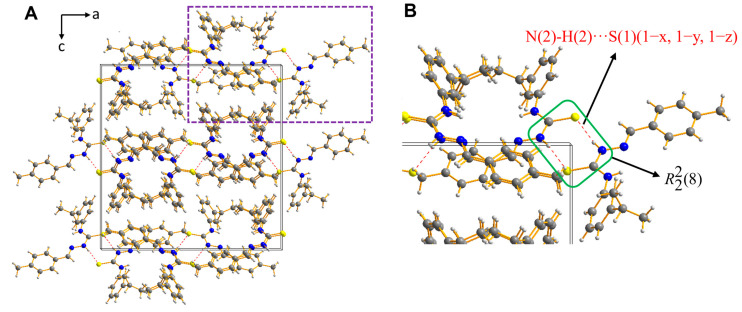
(**A**) Crystal packing diagram of the title compound as viewed along the b-axis. (**B**) Enlargement of the purple box in (**A**). The intermolecular hydrogen bonding is shown by the red dotted lines.

Figure 2 shows the crystal packing diagram of the title compound as viewed along the *b*-axis. Table 3 provides the parameters for intra- and intermolecular hydrogen bonds. As shown in Figure 1A, there was an intramolecular hydrogen bond N(3)-H(3)···N(1) in the title compound, where N(3) acted as the donor atom and N(1) acted as the acceptor atom to build a five-membered ring. Figure 2B, obtained by enlarging the purple box in Figure 2A, clearly indicates the orderly packing of two adjacent title compounds through the $R_{2}^{2}$(8) ring motif consisting of two identical intermolecular hydrogen bonds N(2)-H(2)···S(1) (1 − x, 1 − y, 1 − z), where N(2) acted as the donor and S(1) acted as the acceptor. The findings suggested that intramolecular and intermolecular hydrogen bonding play a role in maintaining the title compound’s crystal structure or packing. Moreover, previous studies have also confirmed that intramolecular hydrogen bonds N... H-N and intermolecular hydrogen bonds C=S... H-N determines the global *syn*-stereochemistry of the molecular architecture [29,30].

### 2.3. Hirshfeld Surface Analysis

**Figure 3:**
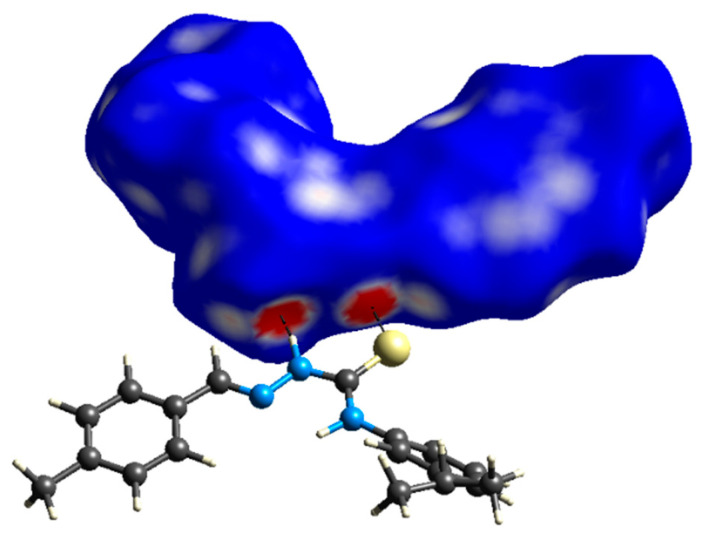
Three-dimensional *d*_norm_ surface of the title compound.

The Hirshfeld surface is a crucial tool for the analysis of molecular crystal structure, which can identify intermolecular interactions. Ghodrat et al. [31] first applied the Hirshfeld surface to study thiosemicarbazones in detail. The Hirshfeld surface is mapped by the *d*_norm_ function to overcome the difficulty that *d_i_* and *d_e_* do not take into account large atoms. The 3D *d*_norm_ surface of the title compound is shown in Figure 3. The plot is displayed using a fixed red-white-blue color scheme (−0.1 Å to 0.4 Å). The regions where contact is shorter than vdW separation (3.27 Å) are filled with red; on the contrary, the regions where contact was greater than vdW separation are filled with blue. There are two equal, dark red dots near N and S atoms in Figure 3, which illustrates that the intermolecular hydrogen bond N(2)-H(2)···S(1) (1 − x, 1 − y, 1 − z) is a strong hydrogen bond interaction. The above analysis confirms that the strong intermolecular hydrogen bond N(2)-H(2)···S(1) (1 − x, 1 − y, 1 − z) existed and played a major role during the crystal packing of the title compound.

**Figure 4:**
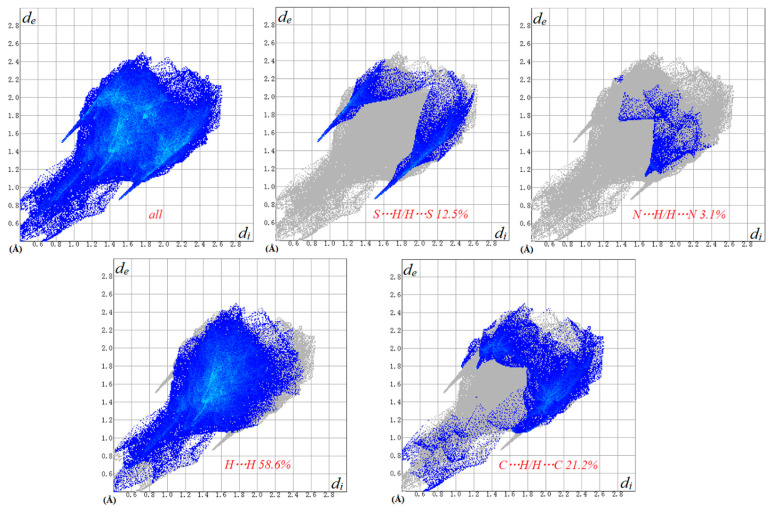
Two-dimensional fingerprint plots of the title compound.

Figure 4 shows 2D fingerprint plots of the main intermolecular interactions of the title compound. The translated 0.6–2.8 Å was used to illustrate the 2D fingerprint plots, which were produced by binning (*d*_i_ + *d*_e_) pairs at intervals of 0.2 Å. The fingerprint plots’ gray areas display the title compound’s overall contacts. The findings show that, with 58.6% of the title compound’s interactions occurring in a cyclic H···H contact, two spikes were seen at the left bottom of the 2D plot (i.e., short *d*_i_ and *d*_e_, with the higher one connected to the donor atom and the lower one to the acceptor). The title compound’s C···H contact (21.2%) is its second primary interaction, with a significantly higher value of (*d*_i_ + *d*_e_). The S···H contact (12.5%) was mainly due to the influence of intermolecular hydrogen bond N(2)-H(2)···S(1) (1 − x, 1 − y, 1 − z), and the value of (*d_i_ + d_e_*) is about 2.4 Å. The short contacts such as C···N (2.1%) and H···N (3.1%) also played a vital role in supporting the crystal packing of the title compound.

### 2.4. AIM Theory

**Figure 5:**
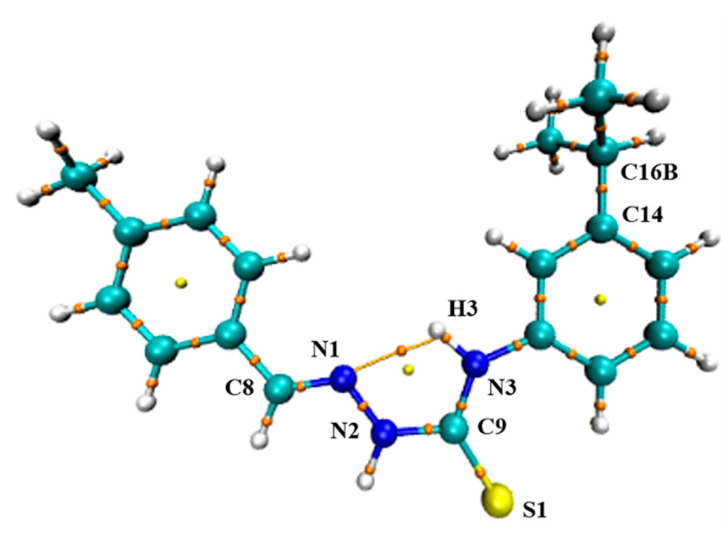
Molecular graph of the title compound computed at the 6-31+G(d,p) basis set with the Multiwfn program. Orange spheres represent bond crucial points, yellow spheres represent ring critical points, and orange lines represent a bond path.

The quantum theory of AIM is useful in the characterization of bonds through a topological analysis of the electronic charge density (*ρ*(BCP)) and their Laplacian (▽^2^*ρ*(BCP)) at the bond critical point (BCP). Moreover, the characteristics of the atoms, which are acquired by integrating the charge density over the atom orbital, and an examination of the charge density’s Laplacian at the BCP can clarify the nature of the bonding relationship [32]. Figure 5 shows the molecular graph of the title compound generated at the 6-31+G(d,p) basis set using the Multiwfn tool. The bond critical point data of the title compound is shown in Table 4. As seen in Table 4, the charge density of the N1=C8, N1-N2, C9-S1, N3-H3, and C14-C16B bond critical points was relatively high and the ▽^2^*ρ* was negative. One can define the regions where charge density is concentrated (▽^2^*ρ* < 0) or depleted (▽^2^*ρ* > 0) in light of the value of Laplacian. The results manifested that the charge density was concentrated in the inter-nuclear regions of chemical bonds N1=C8, N1-N2, C9-S1, N3-H3, and C14-C16B.

Koch et al. [33,34] have proposed criteria based on the AIM theory to explain hydrogen bonds. The energy density at the bond critical point (*H*_BCP_) has also been proven to be a crucial parameter for characterizing the nature of the hydrogen bonds. As shown in Table 4, the value of electron density (0.00232) at the N1···H3 bond critical point was within the reported range (0.002–0.004). Weak hydrogen bond interactions showed both ▽^2^*ρ*(_BCP_) and *H*_BCP_ > 0, and medium hydrogen bond interactions showed ▽^2^*ρ*(_BCP_) > 0 and *H*_BCP_ < 0, while strong hydrogen bond interactions showed ▽^2^*ρ*(_BCP_) and *H*_BCP_ < 0 [35]. The ▽^2^*ρ*(_BCP_) and *H*_BCP_ values at the N1···H3 bond critical point of the title compound were both positive, indicating that the hydrogen bond N1···H3 was a weak intramolecular hydrogen bond interaction. This analysis indicates that the non-covalent intramolecular hydrogen bond N(3)-H(3)···N(1) interaction also played a role in supporting the crystal structure of the title compound, except for the covalent interaction.

### 2.5. ESP Analysis

**Figure 6:**
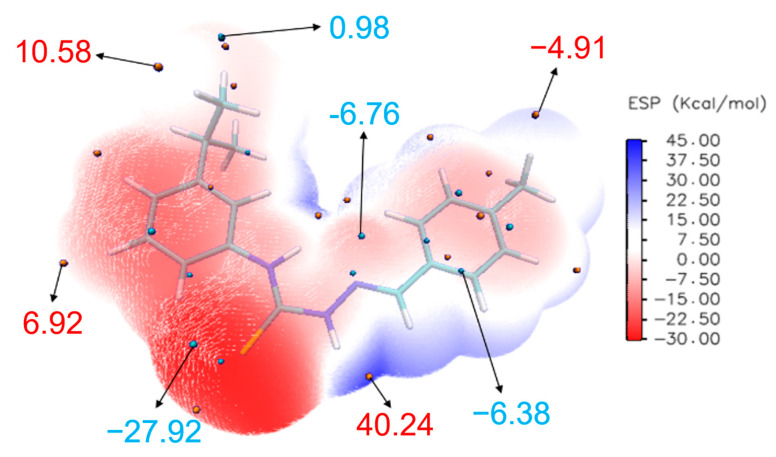
The ESP plot of the title compound.

The electrostatic potential (ESP), which is created by the distribution of charges surrounding a molecule, may be utilized to determine the molecule’s reaction sites. Furthermore, because receptors and ligands identify one another on the molecular surface, the electrostatic potential has a significant impact on the binding sites of these molecules [36,37]. Figure 6 shows the ESP plot of the title compound derived at the B3LYP/6-31+G(d,p) basis set. Furthermore, the local maximum and lowest electrostatic potentials (red and blue dots, respectively) were highlighted. The various colors indicated the values of the electrostatic potential at the ESP surface: the regions of greatest positive, greatest negative, and zero electrostatic potential were represented by the colors red, blue, and white, respectively. Figure 6 illustrates that the title compound’s local maximum electrostatic potential was 40.24 kcal/mol, indicating a significant repulsion of protons by atomic nuclei. In contrast, the title compound’s local lowest electrostatic potential was −27.92 kcal/mol. This suggests that the near regions of the -C=S-NH- group were more susceptible to electrophile attack because of the proton’s attraction to the molecule’s aggregate electron density. In addition, the electrostatic potential in a few white regions in the ESP plot was very nearly zero.

### 2.6. UV-Visible and HOMO-LUMO Analyses

**Figure 7:**
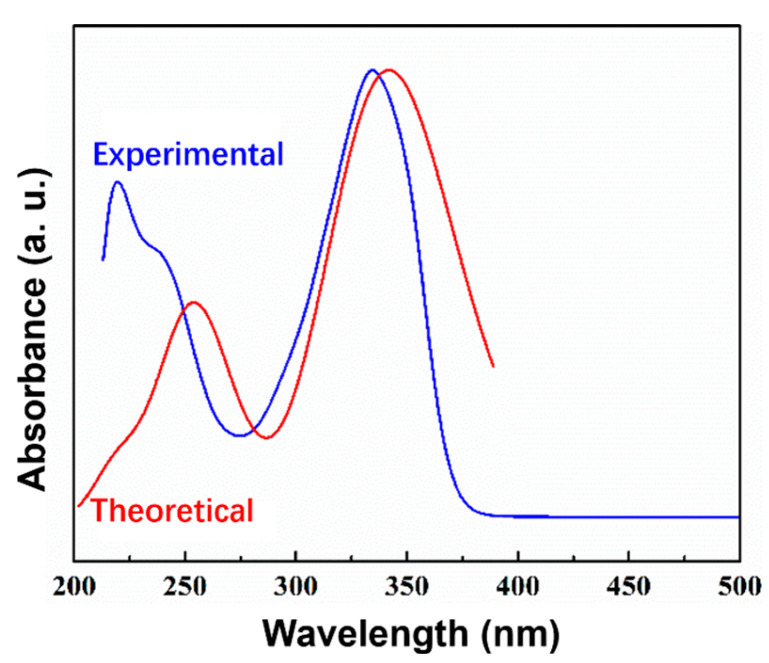
UV-visible absorbance spectrum of the title compound, both theoretical and experimental.

The title compound’s experimental UV-visible absorption spectrum was recorded in the region of 200–500 nm with ethanol as a solvent. Meanwhile, the electronic absorption spectra in ethanol were calculated using the B3LYP/6-31+G(d,p) basis set with TD-DFT. Figure 7 depicts the title compound’s experimental and theoretical UV-visible absorption spectra. The theoretical UV-visible absorption maximum, excitation energy, oscillator strength, and assignment are summarized in Table 5. As shown in Table 5, the theoretical UV-visible absorption maximum values of the title compound were 346 and 259 nm, and the corresponding experimental values were 335 and 220 nm. The oscillator strength *f* was used to describe the strength of electronic transitions within a molecule. For the title compound, the oscillator strength of transition at 346 nm (*f* = 0.95) was higher than the other (*f* = 0.31). The maximum absorption lay at 346 nm was mainly attributable to the electron transition from the highest occupied molecular orbital (HOMO) to the lowest unoccupied molecular orbital (LUMO) and possessed n→π* nature. The absorption band located at 259 nm was mainly assigned to the HOMO-1→LUMO+1 transition, corresponding to an energy difference of 4.79 eV. This transition was predicted to be of π→π* nature. In addition, the excitation energy required for the transition from HOMO to LUMO molecular orbital was lower than the HOMO-1→LUMO+1 transition.

**Figure 8:**
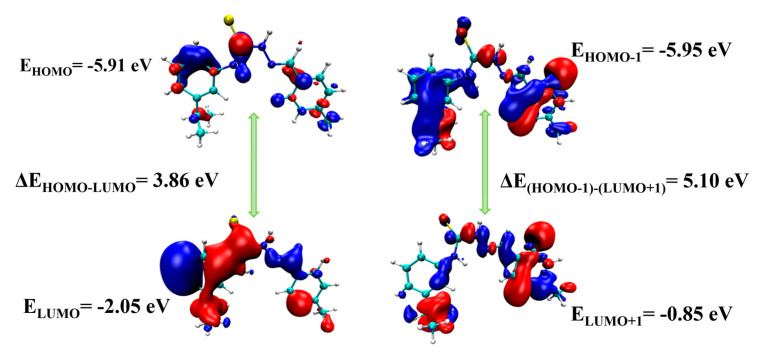
Frontier molecular orbitals of the title compound calculated at the 6-31+G(d,p) basis set.

The HOMO energy, the LUMO energy, and the energy gap between the HOMO and LUMO molecular orbitals are all required to forecast molecule energetic behavior [38]. The HOMO represents electron donation, the LUMO represents electron acceptance, and the Δ*E*_HOMO-LUMO_ energy gap reflects the kinetic stability and chemical reactivity of molecules [37]. Figure 8 exhibits the frontier molecular orbitals of the title compound calculated at the 6-31+G(d,p) basis set. The HOMO orbital was primarily composed of C9, C11, C5, and C1 atoms, and the LUMO orbital spread over the entire molecule. The HOMO-1 and LUMO+1 molecular orbitals are related to the electronic transitions of the title compound. The HOMO-1 orbital spreads over the entire molecule, and the LUMO+1 orbital mainly spreads over the central skeleton (=N-N-C(S)-N-) and the benzene ring, linking with the methyl group. The energy values of HOMO and LUMO orbitals and Δ*E*_HOMO–LUMO_ and Δ*E*_HOMO-1–LUMO+1_ energy gaps are illustrated in Figure 8. The Δ*E*_HOMO–LUMO_ energy gap of the title compound was theoretically calculated to be 3.86 eV. This value was utilized to describe the eventual charge transfer interaction with the title compound, which had an impact on its biological activity. Generally speaking, a larger Δ*E*_HOMO–LUMO_ energy gap implies high kinetic stability and low chemical reactivity [39]. A similar value of Δ*E*_HOMO–LUMO_ energy gap (3.87 eV) reported by Gil implied that the reported compound was reactive and, hence, less stable [40]. Therefore, the low Δ*E*_HOMO–LUMO_ energy gap indicates that the title compound is reactive.

### 2.7. Molecular Docking

**Figure 9:**
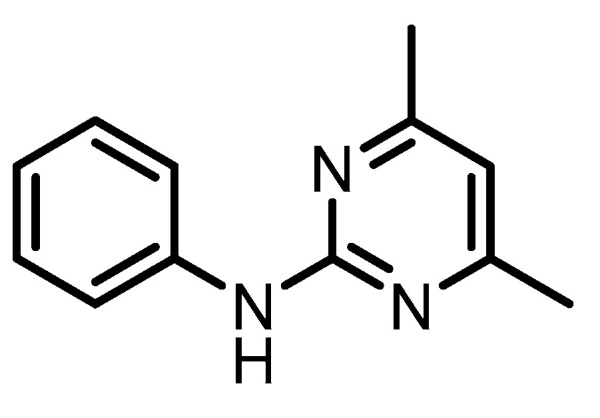
The structural formula of *pyrimethanil*.

The concepts of energy matching, geometric matching, and chemical environment matching were employed in molecular docking to evaluate binding ability and forecast the optimal combination mode. This method is useful for visualizing protein-ligand interactions [41]. *N*-myristoyltransferase (PBD ID: 1NMT) is a cytosolic monomer enzyme that catalyzes the transfer of myristate from myristoyl-CoA to a subset of eukaryotic cellular proteins’ *N*-terminal GLY residues [42]. NMTs have been well investigated in *S. cerevisiae* and human cells and are required for pathogenic fungal viability [43]. As a result, 1NMT has been identified as the target enzyme for a variety of antifungal medication research efforts. In this study, a standard drug (*pyrimethanil*) was utilized to compare with the title compound, and its structural formula was presented in Figure 9. The docking parameters were calculated using AutoDockTools-1.5.6 software, and Discovery Studio 3.0 software was used to visually display the interactions of the functional isomer of the title compound and *pyrimethanil* with the receptor protein. The docking results are shown in Table S1, including docking energy, inhibition constant Ki, and residue of the active site.

**Figure 10:**
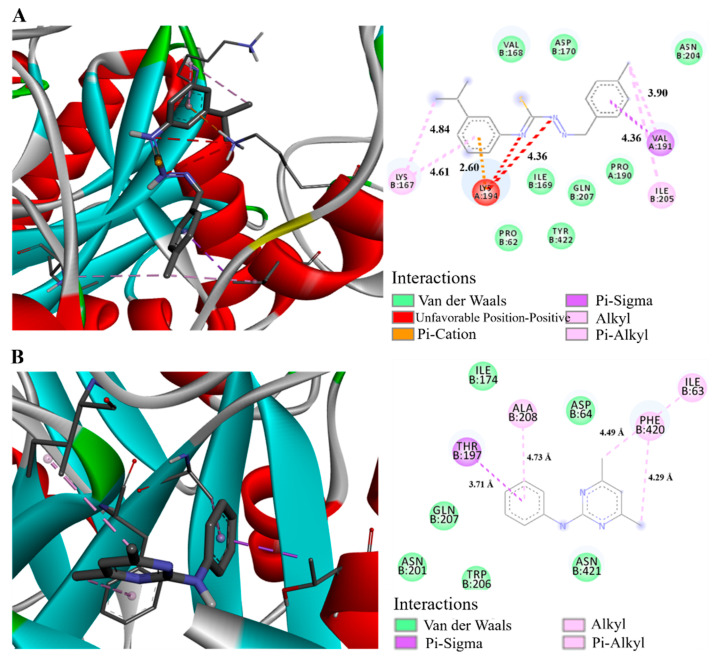
Three-dimensional (**left**) and two-dimensional (**right**) docking modes of the functional isomer of the title compound (**A**) and *pyrimethanil* (**B**) with the receptor protein (1NMT).

As shown in Figure 10, there were residues VAL168; ASP170; ASN204; LYS167; LYS194; ILE169; GLN207; PRO190; VAL191; ILE205; PRO62; TYR422; ASN201; THR197; ILE174; ALA208; TRP206; ASP64; ASN421; PHE420; and ILE63 around the active pocket, which bound with the functional isomer of the title compound (Figure 10A) and *pyrimethanil* (Figure 10B) through multiple interactions, such as Pi-Alkyl, van der Waals force, Pi-Sigma, and so on. The docking energies of the title compound and *pyrimethanil* with the receptor protein (1NMT) are −4.76 and −4.28 Kcal/mol, respectively. The title compound showed lower docking energy and higher affinity with the receptor protein (1NMT) compared with *pyrimethanil*. The findings suggest that the title compound could become a better candidate for antifungal medicines.

### 2.8. Antifungal Activity

**Figure 11:**
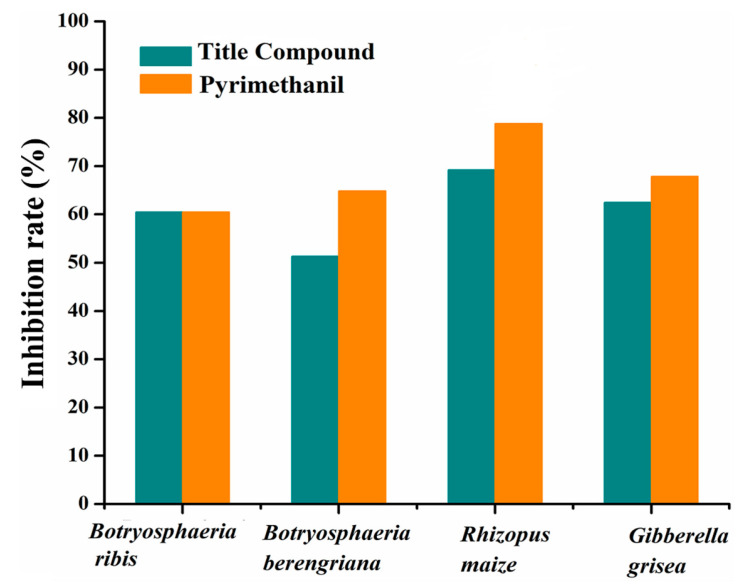
Antifungal activity of the title compound and *pyrimethanil*.

Figure 11 and Table S2 illustrate the antifungal activity of the target drug and *pyrimethanil* against four fungal strains: *Gibberella grisea*, *Rhizopus maize*, *Botryosphaeria ribis*, and *Botryosphaeria berengriana*. The findings demonstrate that the title compound’s inhibition rates are 59.46%, 51.77%, 68.95%, and 62.50%, respectively, against *Botryosphaeria ribis*, *Botryosphaeria berengriana*, *Rhizopus maize*, and *Gibberella grisea*. The inhibition activity of the title compound against *Rhizopus maize* is most remarkable therein. Except for strain *Botryosphaeria ribis*, the title compound’s inhibition rates against the other three strains are marginally lower than those of *pyrimethanil*. Particularly, the difference in inhibition rate for strain *Rhizopus maize* was 8.42%. It is worthwhile noting that the title compound shows a similar behavior with *pyrimethanil* for strain *Botryosphaeria ribis*. Combined with the molecular docking analysis, the title compound may be a viable contender for antifungal drugs in the future.

## 3. Experimental

### 3.1. General

All chemicals (reagents and solvents) were of analytical grade and were utilized without additional purification. The synthesis was realized in a BILON-CW-1000 microwave synthesizer at a suitable absorption power 800 W setting. The melting point was determined on a Cossim KER3100-08S apparatus and was not corrected. Thin-layer chromatography (TLC) was carried out on an ultraviolet analyzer for visualization to monitor the progress of the reaction. Elemental analysis was performed using an ELEMENTAR Vario ELIII instrument. FT-IR spectra were recorded on an EQUINOX 55 FT-IR spectroscope in the range 4000**–**500 cm^−1^. NMR spectra were performed on a Bruker 353 instrument operating at 400 and 100 MHz for ^1^H and ^13^C nuclei, respectively. All chemical shifts (*δ*_H_, *δ*_C_ values) are given in parts per million (ppm); all homocoupling patterns (^n^*J*_H,H_ values) are given in hertz (Hz). Multiplicities (signals splitting) in the reported ^1^H NMR spectrum are indicated as s = singlet, d = doublet, t = triplet, m = multiplet, and h = heptet. No TMS was added: chemical shifts *δ*_H_ and *δ*_C_ were measured against the solvent peak (DMSO-*d*_6_) taken as a reference signal (*δ*_H_ = 2.50 ppm, *δ*_C_ = 39.52 ppm). The UV-Vis spectrum was acquired in ethanol using a SHIMADZU UV-2600 spectrophotometer equipped with quart cells. The single-crystal X-ray data were obtained using a Bruker APEX-II CCD diffractometer.

### 3.2. One-Pot Four-Step Synthesis of (E)-1-(4-Methylbenzylidene)-4-(3-Isopropylphenyl) Thiosemi-Carbazone ***4*** (Scheme 1)

In a BILON-CW-1000 microwave synthesizer (800 W) set at 25 °C, an ethanol (30 mL) solution containing 3-isopropylaniline (1.2 g, 1.2 mL, 10 mmol), aq. NH_3_ 25% (1.9 g, 2.1 mL, 50 mmol), and carbon disulfide (1.3 g, 1.0 mL, 10 mmol) were stirred at room temperature for 2 h until the 3-isopropylaniline was completely consumed through TCL (R_f 3-isopropylaniline_ = 0.2 (PE/EtOAc, (1/2 *v*/*v*)). Then, sodium chloroacetate solid (1.1648 g, 10 mmol) was added, and the reaction mixture was stirred for an additional 2 h until compound **1** was completely disappeared by TCL (R_f compound **1**_ = 0.21 (PE/EtOAc, (1/4 *v*/*v*)). Next, 95% hydrazine hydrate (1.3 g, 1.3 mL, 10 mmol) was added and the reaction mixture was stirred for 2 h until compound **2** completely disappeared by TCL (R_f compound_ **_2_** = 0.26 (MeOH/Cl_2_Cl_2_/AcOH, (100/100/1 *v*/*v*/*v*)). Finally, 4-methylbenzaldehyde (1.2 g, 1.2 mL, 1.0 mmol) was added, and the reaction mixture, as a solution, was stirred at room temperature for about 2 h until complete depletion of compound **3** was confirmed by TCL (R_f compound **3**_ = 0.25 (PE/EtOAc/Et_3_N, (100/100/1 *v*/*v*/*v*)), while the desired white-colored compound **4** was precipitated, separated by the filtration, and dried. This product was recrystallized from ethanol.

### 3.3. Analytical Data

White powder solid 1.35 g (43.4% global yield, with respect to the theoretical mass of title compound). M.p. = 135.5–136.8 °C (diphenyl ether). R_f_ = 0.35 (PE/EtOAc, (2/1 *v*/*v*), visualization in 254 nm). Elemental anal. (%) calcd. for C_18_H_21_N_3_S (found): C 69.42(69.02), H 6.8(7.00), N 13.49(13.69). FT-IR (KBr) **ν**max 3318 (NH, CN-H), 3132 (NH, NN-H), 2948 (C-H), 1640 (C=N), 1385**–**1365 (CH_3_), 1250**–**1200 (C-N), 727 (C=S) cm**^−^**^1^. ^1^H NMR (400 MHz, DMSO-*d*_6_) *δ*_H_, 11.76 (s,1H, NNH), 10.04 (s, 1H, NH), 8.14 (s, 1H, CH=N), 7.80 (d, *J* = 8.0 Hz, 2H, Ph), 7.44 (s, 2H, Ph), 7.30**–**7.22 (m, 3H, Ph), 7.08 (d, *J* = 7.6 Hz, 1H, Ph), 2.90 (hept, *J* = 6.8 Hz, 1H, CH), 2.34 (s, 3H, CH_3_), 1.23 (d, *J* = 6.9 Hz, 6H, (CH_3_)_2_) ppm. ^13^C NMR (100 MHz, DMSO-*d*_6_) *δ*_C_, 21.5 (CH_3_), 24.3 ((CH_3_)_2_), 33.8 (CH), 123.6**–**140.3 (Ph), 143.4 (C=N), 148.8 (Ph-CH(CH_3_)_2_), 176.1 (C=S) ppm.

### 3.4. Crystallographic Data Collection and Refinement

Using a slow evaporation approach, a petroleum solution of the title compound was allowed to come to room temperature for a week before being crystallized and used for X-ray diffraction utilizing Mo *K*α radiation (*λ* = 0.071073 nm). The direct approach with SHELXS 97 was used to tackle the structure refinement problem [44]. The non-hydrogen atoms were refined using SHELXS 97 in the full-matrix-block least-squares approach on *F*^2^ with anisotropic thermal parameters [45]. The riding concept was followed with the addition of hydrogen atoms. The additional crystallographic data was included in Deposition Number 1,971,480 and was made available without charge by the joint Cambridge Crystallographic Data Centre. Diamond 3.2k software was utilized to construct the packing diagrams and crystal structure, while the PLATON tool was employed to calculate the extra data.

### 3.5. Quantum Chemical Calculation

One of the most crucial phases in theoretical computations is geometry optimization. The title compound’s X-ray diffraction data was utilized to optimize the structure. Equations for density functional theory (DFT) were computed with Gaussian 09W and the Multiwfn program. The 6-31+G(d,p) basis set and Becke’s three-parameter hybrid exchange functionals with the Lee–Parr correlation functionals (B3LYP) approach were selected [46]. Software tools Gauss View 5.0 and VMD1.9.1 were used to display the plots. Additionally, the CrystalExplorer3.1 software package was used to create 2D fingerprint plots and 3D *d*_norm_ surface representations of the target molecule [47].

### 3.6. Antifungal Assay

Using the disc diffusion method, the title compound’s antifungal activity was investigated. Dimethyl sulfoxide (DMSO) was used to dissolve the title compound, and the resulting mixture was then transferred into sterile PDA to create a solution with a concentration of 100 mg/L. All of the Petri dishes were injected with the preactivated fungi in a sterile setting. The Petri plates were then put in an incubator to ensure that the mycelium would continue to grow upward. Following a 72-h incubation period at 25 °C, the disc diffusion diameter was determined. To lower experimental errors, three sets of parallel tests were carried out in the interim [48]. Marketable *pyrimethanil* was utilized as a positive control, and a sterile petri dish containing only the PDA culture media was employed as a blank control. The inhibition rate was determined using the Formula (1):

(1) $$\mathrm{Inhibition}\;\mathrm{rate}\;(\%)=\frac{D_{b}-D_{t}}{D_{b}-8}\;\times \;100\%$$

where *D_b_* and *D_t_* were the diameters of blank and test, respectively.

## 4. Conclusions

In summary, the novel (*E*)-1-(4-methylbenzylidene)-4-(3-isopropylphenyl) thiosemicarbazone was prepared and its structure was validated using various characterization techniques, such as FTIR, NMR, and single-crystal X-ray diffraction techniques, etc. Single-crystal investigation revealed that the intramolecular hydrogen bond N(3)-H(3)···N(1) and intermolecular hydrogen bond N(2)-H(2)···S(1) (1 − x, 1 − y, 1 − z) existed in the crystal structure and packing of the title compound. AIM theory suggested that the title compound’s crystal structure was also maintained via the weak non-covalent intramolecular hydrogen bond N(3)-H(3)···N(1), except for the covalent interaction. Furthermore, Hirshfeld surface analysis found that the strong intermolecular hydrogen bond N(2)-H(2)···S(1) (1 − x, 1 − y, 1 − z) contributed significantly to the crystal packing of the title compound. Regions near the C=S bond displayed the local minimum electrostatic potential, making them susceptible to electrophilic attacks. According to UV-visible spectra and HOMO-LUMO analysis, the transition from the HOMO to LUMO molecular orbital belongs to n→π* nature. The low *ΔE*_HOMO_–_LUMO_ energy gap (3.86 eV) suggested that the title compound has high chemical reactivity and potential antifungal activity. Molecular docking studies indicated that the target chemical has a greater affinity for the antifungal receptor protein (1NMT) than *pyrimethanil*. For the strain *Botryosphaeria ribis*, the title compound exhibited antifungal effects similar to those of *pyrimethanil*. As a consequence, the title compound designed in the current study can be considered as a potential candidate for antifungal drugs in the future.

## Figures and Tables

**Table 1:** Data collection and structure refinement details of the title compound.

Chemical Formula	C_18_H_21_N_3_S
Formula weight	311.44
Temperature (K)	296 (2)
Wavelength (Å)	0.71073
*a* (Å)	20.224 (4)
*b* (Å)	8.4969 (15)
*c* (Å)	20.602 (4)
*α* (degree)	90.00
*β* (degree)	90.00
*γ* (degree)	90.00
Volume (Å^3^)	3540.3 (12)
*Z*	8
*D*_c_ (Mg/m^3^)	1.169
Crystal system	Orthorhombic
Space group	Pbca
Index ranges	−23 < *h* < 23*,* −5 < *k* < 10, −24 < *l* < 24
*F* (000)	1328
Theta range for data collection (°)	2.2 to 25.0
Reflections collected	16947
Independent reflections	3114
*R*_int_	0.108
Goodness-of-fit on *F*^2^ (S)	0.886
*R* (all data)	*R*_1_ = 0.0670, *wR*_2_ = 0.2188

**Table 2:** Selected experimental and theoretical geometrical parameters of the title compound at the B3LYP/6-31+G(d,p) basis set.

Parameters	Experimental	Calculated
Bond length (Å)		
S(1)-C(9)	1.668	1.668
N(1)-C(8)	1.269	1.289
N(2)-C(9)	1.357	1.357
N(3)-C(9)	1.336	1.355
C(2)-C(3)	1.362	1.345
C(1)-C(2)	1.515	1.510
N(3)-C(10)	1.417	1.413
Bond angle (°)		
C(17B)-C(16B)-C(18B)	123.0	124.5
C(13)-C(14)-C(16B)	119.1	120.9
C(4)-C(5)-C(8)	119.9	120.0
C(3)-C(2)-C(7)	117.4	117.9
S(1)-C(9)-N(3)	126.2	126.0
Dihedral angle (°)		
C(8)-N(1)-N(2)-C(9)	−179.0	0.6
N(1)-N(2)-C(9)-S(1)	−177.3	−177.3
N(1)-N(2)-C(9)-N(3)	3.9	3.6
C(10)-N(3)-C(9)-S(1)	0.8	1.0

**Table 3:** Hydrogen bonds of the title compound.

D-H…A	*d*(D-H)	*d*(H…A)	*d*(D…A)	∠D-H…A
N(3)-H(3)···N(1)	0.8600 Å	2.1800 Å	2.618(5) Å	117.000°
N(2)-H(2)···S(1) ^*a*^	0.8600 Å	2.5200 Å	3.372(3) Å	171.000°

^[*a*]^ 1 − x, 1 − y, 1 − z.

**Table 4:** Bond critical point information of the title compound calculated at the 6-31+G(d,p) basis set.

Bond (X-Y)	*ρ*	▽^2^*ρ*_(BCP)_	*H*_BCP_	Position (*x*, *y*, *z*)
N1=C8	0.00366	−0.00505	−0.00625	(−4.17751, −3.09275, 0.00007)
N1-N2	0.00361	−0.00695	−0.00359	(−1.84763, −3.81886, 0.00008)
C9-S1	0.00210	−0.03291	−0.00247	(2.57242, −6.32864, 0.00013)
N3-H3	0.00341	−0.01834	−0.00510	(1.28564, −1.27543, −0.00007)
C14-C16B	0.00250	−0.00583	−0.00203	(7.15390, 4.32850, −0.00006)
Interaction N1···H3	0.00232	0.08696	0.00198	(−0.39692, −1.75043, −0.00003)

**Table 5:** Theoretical electronic absorbance spectrum of the title compound at the TD-DFT/B3LYP/6-31+G(d,p) basis set.

Excited State	Wavelength (nm)		Excitation Energy (eV)	Oscillator Strength (*f*)	Assignment
	Theoretical	Experimental			
S1	346	335	3.58	0.95	HOMO→LUMO (67%)
S2	259	220	4.78	0.31	HOMO-1→LUMO+1 (65%)

## Data Availability

Data are contained within the article.

## Supplementary Materials

The following supporting information can be downloaded at: https://www.mdpi.com/article/10.3390/molecules29194702/s1, Figure S1: The FTIR spectrum of the title compound; Figure S2: The ^1^H NMR spectrum of the title compound.; Figure S3: The ^13^C NMR spectrum of the title compound. Table S1: Docking energy, inhibition constant Ki, and residue of the active site for the title compound and *pyrimethanil*; Table S2: Antifungal activity of the title compound and *pyrimethanil*.
