# Supplementary material for: Synthesis, Crystal Structure and Antifungal Activity of (E)-1-(4-Methylbenzylidene)-4-(3-Isopropylphenyl) Thiosemicarbazone: Quantum Chemical and Experimental Studies
Source: Molecules. 2024 Oct 4;29(19):4702. doi: 10.3390/molecules29194702 (PMC11477955; doi:10.3390/molecules29194702)
Supplement: Supplementary file 1 [file molecules-29-04702-s001.zip › molecules-3204249-supplementary.pdf]

Supplementary Materials

Synthesis, Crystal Structure and Antifungal Activity of (*E*)-1-(4-Methylbenzylidene)-4-(3-isopropylphenyl) Thiosemicarbazone: Quantum Chemical and Experimental Studies

Haitao Ren [1,*,†], Fan Qi [2,†], Yuzhen Zhao [1], Abdelkader Labidi [3] and Zongcheng Miao [1,4,*]

[1] Technological Institute of Materials & Energy Science (TIMES), Xijing University, Xi'an 710123, China
[2] State Key Laboratory of Medicinal Chemical, College of Pharmacy, Nankai University, Tianjin 300071, China
[3] School of Environmental Science and Engineering, Shaanxi University of Science and Technology, Xi'an 710021, China
[4] School of Artificial Intelligence, Optics and Electronics (iOPEN), Northwestern Polytechnical University, Xi'an 710072, China
* Correspondence: bs210311007@sust.edu.cn (H.R.); miaozongcheng@nwpu.edu.cn (Z.M.)
† These authors contributed equally to this work.

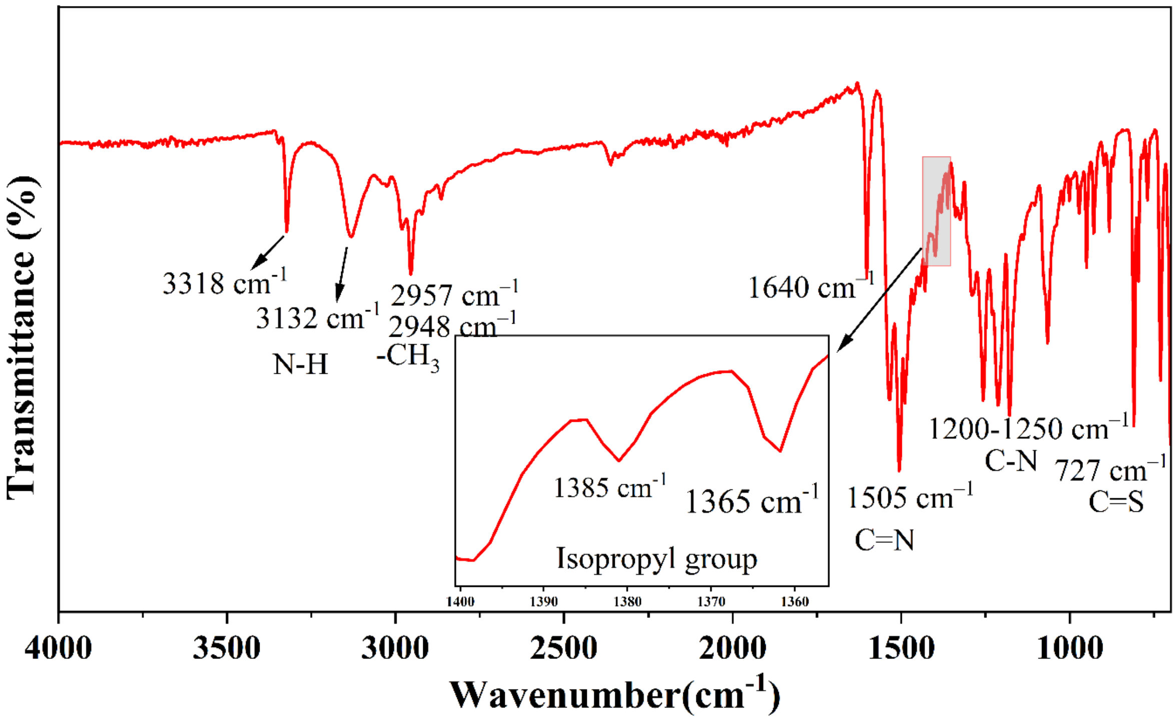

Figure S1. The FTIR spectrum of the title compound.

The FTIR spectrum of the title compound, which is shown in Figure S1, was recorded in the 4000-500 cm^{-1} range. Medium-strong peaks at 3318 and 3132 cm^{-1} in the FTIR spectrum is classified into the N-H stretching vibration. CH_3 group symmetric stretching vibration lay in 2948 and 2957 cm^{-1}. The isopropyl group induced the coupling split, resulting in the appearance of the two weak peaks with similar intensities near 1385 and 1365 cm^{-1}. C=N stretching vibration located at 1604 cm^{-1} in the FTIR spectrum is slightly different from the reported values (1630-1690 cm^{-1}) and occurred the redshift, probably due to the conjugation action of the C=N bond and benzene ring. The peak at 727 cm^{-1} in the FTIR spectrum represents C=S in-plane bending vibrations.

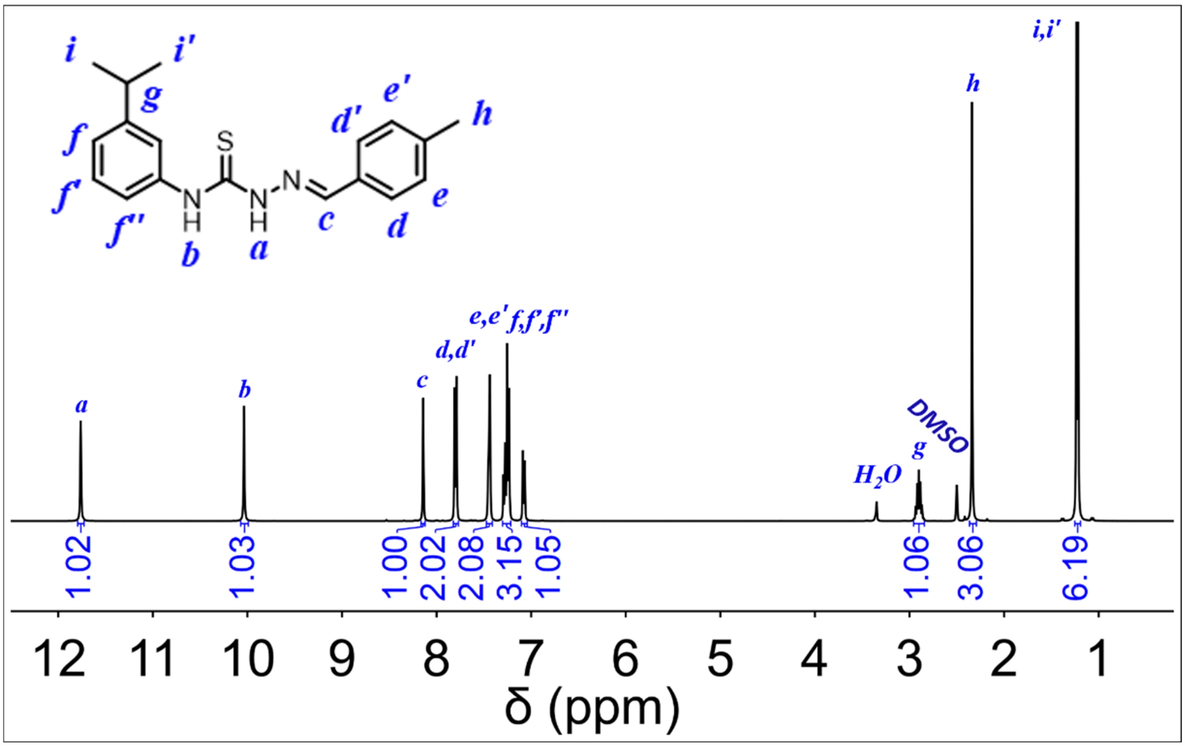

Figure S2. The ^{1}H NMR spectrum of the title compound.

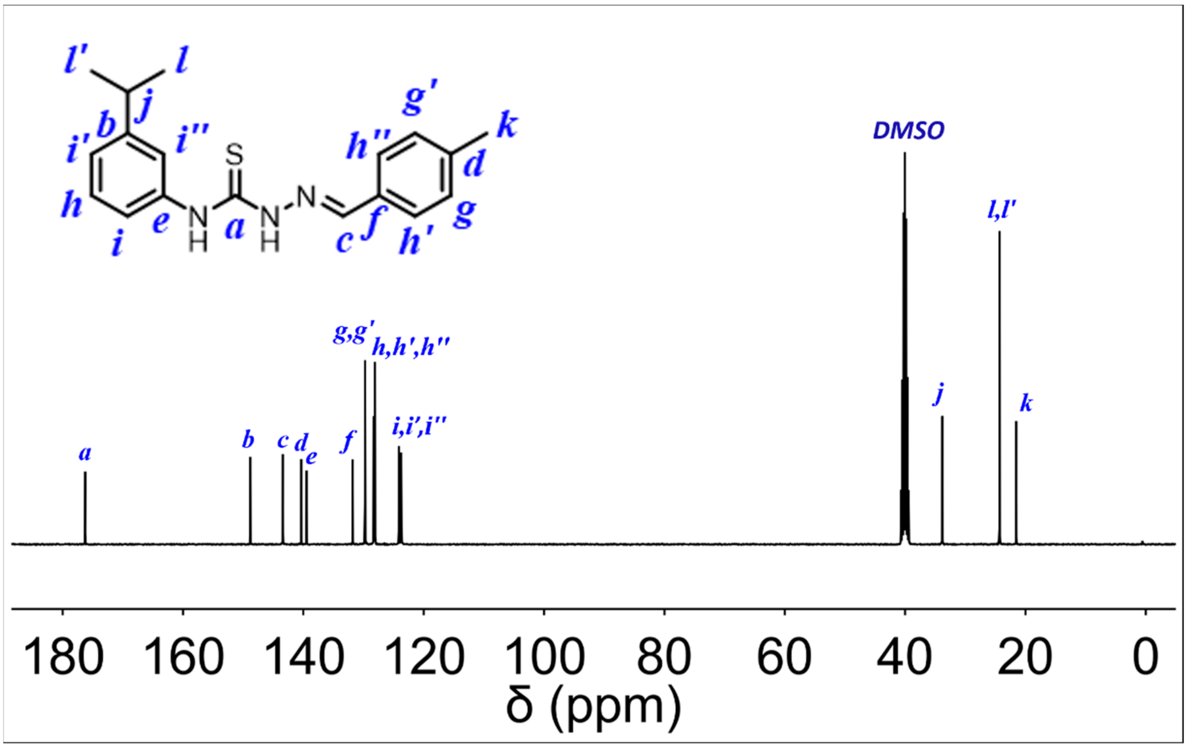

Figure S3. The ^{13}C NMR spectrum of the title compound.

Figure S2 shows the ^{1}H NMR spectrum of the title compound. The signals that appeared at 11.77 and 10.03 ppm are assigned to CN-H and NN-H protons, respectively. The signal located at 8.13 ppm in the 1H NMR spectrum is attributed to CH=N proton. The protons on -$CHMe_2$ group situated at 2.89 ppm. The protons

of -CH3 group on the benzene ring of the title compound located at 2.33 ppm. The signal situated in 1.23 ppm represented the six protons with the same chemical environment, which is assigned to the protons in the isopropyl group. Besides, the ^{13}C NMR spectrum of the title compound is displayed in Figure S3. C=S and C=N peaks of the title compound are located at 176.20 and 148.80 ppm respectively. The signals in 143.32-123.61 ppm are assigned to carbons on the benzene ring. The -CH and -Me carbons on $-CHMe_2$ group situated at 33.72 and 24.10 ppm in sequence. The methyl carbon on benzene ring of the title compound is located at 21.43 ppm.

Table S1. Docking energy, inhibition constant Ki, and residue of active site for the title compound and *pyrimethanil*.

1NMT	Docking Energy (kcal/mol)	Ki (μmol/L)	The Residue of the Active Site
Target compound	-4.76	322.17	VAL168; ASP170; ASN204; LYS167; LYS194; ILE169; GLN207; PRO190; VAL191; ILE205; PRO62; TYR422
Pyrimethanil	-4.28	732.56	ASN201; GLN207; THR197; ILE174; ALA208; TRP206; ASP64; ASN421; PHE420; ILE63

Table S2. Antifungal activity of the title compound and *pyrimethanil*.

Compound	*Botryosphaeria ribis*		*Botryosphaeria berengriana*		*Rhizopus maize*		*Gibberella grisea*	
	AD/mm	IR/%	AD/mm	IR/%	AD/mm	IR/%	AD/mm	IR/%
Title compound	23	59.46	26	51.77	16	68.95	29	62.50
Blank	45	-	34	-	46	-	64	-
Pyrimethanil	23	59.46	21	66.53	9	77.37	26	67.86

Note: AD is the average diameter of the disc diffusion, and IR is the inhibition rate.
